# Large nearshore storm waves off the Irish coast

**DOI:** 10.1038/s41598-019-51706-8

**Published:** 2019-10-28

**Authors:** Francesco Fedele, James Herterich, Aziz Tayfun, Frederic Dias

**Affiliations:** 1 School of Civil & Environmental Engineering, Georgia Institute of Technology, Atlanta, Georgia 30332 USA; 2 University College Dublin, School of Mathematics and Statistics, Earth Institute, Belfield, Dublin 4 Ireland; 3 Burak Sitesi 75/1, Bitez, Bodrum, 48400 Mugla, Turkey; 4 CMLA, ENS Paris-Saclay, CNRS, Université Paris-Saclay, 94235 Cachan, France

**Keywords:** Physical oceanography, Fluid dynamics

## Abstract

We present a statistical analysis of nearshore waves observed during two major North–East Atlantic storms in 2015 and 2017. Surface elevations were measured with a 5-beam acoustic Doppler current profiler (ADCP) at relatively shallow waters off the west coast of Ireland. To compensate for the significant variability of both sea states in time, we consider a novel approach for analyzing the non-stationary surface-elevation series and compare the distributions of crest and wave heights observed with theoretical predictions based on the Forristall, Tayfun and Boccotti models. In particular, the latter two models have been largely applied to and validated for deep-water waves. We show here that they also describe well the characteristics of waves observed in relatively shallow waters. The largest nearshore waves observed during the two storms do not exceed the rogue thresholds as the Draupner, Andrea, Killard or El Faro rogue waves do in intermediate or deep-water depths. Nevertheless, our analysis reveals that modulational instabilities are ineffective, third-order resonances negligible and the largest waves observed here have characteristics quite similar to those displayed by rogue waves for which second order bound nonlinearities are the principal factor that enhances the linear dispersive focusing of extreme waves.

## Introduction

Recent studies^1,2^ reveal that rogue waves can arise from a combination of the process of constructive interference and nonlinear effects specific to the complex dynamics of ocean waves. Under relatively rare conditions, waves locally propagate in an organized way or nearly in phase, resulting in an unusual case of constructive interference that generates waves with large amplitudes. However, this mechanism still cannot fully explain the sizes of rogue waves observed under actual oceanic conditions. Various discrepancies observed between theoretical models and actual observations can be attributed to the nonlinear nature of waves: they are not sinusoidal but vertically asymmetric, displaying shallower more rounded troughs, and higher sharper crests that result from the water surface being pushed upward against the pull of gravity. Thus, the nonlinearity of the ocean surface manifest in the lack of symmetry between wave crests and troughs needs to be accounted for^3–5^. Such nonlinearities do contribute to the effects of constructive interference noticeably. Indeed, recent studies^1,4^ suggest that nonlinear effects due to second-order bound harmonics play a predominate role in this process and can cause an increase of 15 to 20 percent in crest height, i.e. the vertical distance from the mean sea level to the top of the wave.

The formation of a rogue wave at a given point of the ocean is simply a random or chance event^1^. Several cases of extreme wave occurrences of practical and theoretical interest such as the Andrea, Draupner and Killard waves^1^ and the sinking of El Faro^2^ have been studied in detail by way of higher order spectral wave simulations and validated with probabilistic wave models. These studies have shown that second-order statistical distributions of crests, in particular those often referred to as Tayfun^3,4,6,7^ and Forristall^8^ models, both describe rogue statistics reasonably well in intermediate to deep waters.

In this work, we will show that the Tayfun and Boccotti^9,10^ models for wave heights, previously validated for both simple^4,10^ and mixed seas^11–13^ in deep water, describe the statistics of large waves in intermediate to relatively shallow waters reasonably well also. For comparison, we also consider the Forristall’s Weibull regression model^8^ because of its frequent application and popularity in engineering design^14^. Our results here and several others elsewhere indicate that it does work quite well in describing the distribution of observed data. For example, Gibson *et al*.^14^ use the Forristall’s model to explain their statistics of oceanic wave crests, but they do not use the Tayfun^3^ or Tayfun-Fedele^4^ models on the grounds that they require the calculation of a key parameter from the time trace of water surface elevations, not readily available from hindcast models. However, they were able to use the Boccotti^9^ wave-height model with two parameters specifically dependent on the frequency spectrum or the time trace of surface elevations. The Forristall crest-height model likewise requires two parameters that depend on the spectral moments. Similarly, recent work by Katsardi *et al*.^15^ indicates that various regression models fitted to observed data are not universally applicable nor do they provide an adequate description of waves in shallow water, as large waves are overestimated. However, only unidirectional laboratory waves propagating on rather mild impermeable slopes are explored, and neither the Tayfun crest-height model^3,4^ nor the Boccotti wave-height models^9,10^ are tested on the grounds that second-order models cannot describe highly nonlinear shallow-water waves affected by intense wave-breaking. Nonetheless, they consider the Forristall crest-height model in their comparisons. That model is also second-order, and all second-order models break down in shallow water where waves are highly nonlinear, prone to intense breaking and affected by various dissipative effects of the seabed. Nevertheless, all these models are equally applicable to some of their data representative of the relatively shallower waters of the transitional water depths.

**Figure 1:**
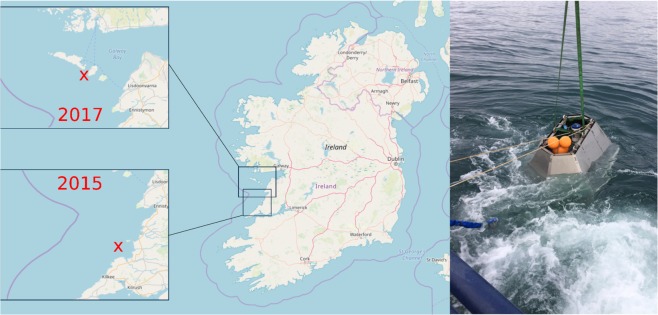
(Left) Map of Ireland: ADCP location off the Aran Islands in 2017 (upper Inset) and off Killard Point in 2015 (lower Inset). (Right) ADCP deployment with (blue-capped) instruments in a protective frame. The map was generated from data via OpenStreetMap and its contributors. Photo by F. Dias.

**Figure 2:**
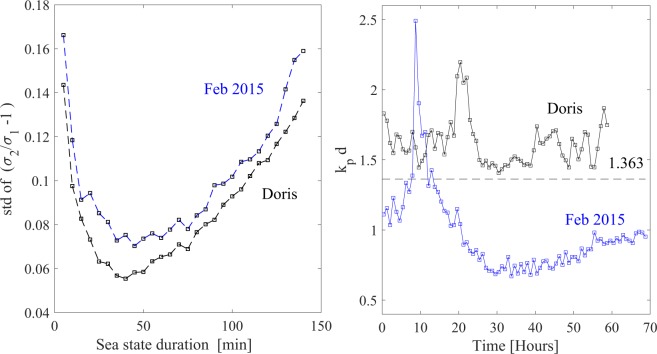
(Left) Variations of difference between consecutive sea states of Doris and Feb 2015 storms, measured in terms of the standard deviation of observed values of *σ*_2_/*σ*_1_ − 1 between two consecutive sea states in a storm sequence. (Right) Hourly variations of the depth factor *k*_*p*_*d* and (dashed line) theoretical threshold above which plane waves are modulationally unstable in unidirectional seas^30,31^.

In the present study, we consider all the aforementioned models and test them against directional waves observed in relatively shallow waters within the shoaling zone. In particular, we consider two wave data sets from ADCP measurements taken off the west-coast of Ireland near Killard Point in 2015 and near the Aran Islands in 2017 (see Fig. 1). In particular, the two locations are nearshore at a water depth of approximately 37 meters (Killard) and 45 meters (Aran). They are well-known high-energy coastlines where storm waves overtop cliffs, fracture bedrock, and move large rocks weighing 100 tons or more^16–19^. We then analyze wave statistics in relatively shallow waters during storm events. In particular, we examine data observed in two storms, namely the storm of 25–27 Feb 2015, hereafter referred to as Feb 2015, and Doris of 21–26 Feb 2017. Wave measurements carried out during these storms are described in the Methods section. Monochromatic waves propagating on a water depth *d* and characterized with a wave number *k* feel the presence of the bottom, and start being modified whenever the dimensionless depth parameter *kd* < *π*. The wave regime is classified as deep water if *kd* > *π*, as intermediate or transitional depth for *π*/10 < *kd* < *π*, and as shallow water if *kd* < *π*/10. This classification has significance both practically and theoretically in establishing how wave characteristics are modified and what processes need be included and modeled in their theoretical predictions. Obviously, defining the depth regime of a wind-wave field as a whole in a similarly precise fashion is impossible due to the wide range of wave numbers observed. As a compromise, we will define the depth regime for the two storms based on the dominant wavenumber *k*_*p*_ at the spectral peak, thus focusing our attention on the most energetic components with wave numbers at and near the spectral peak. On this basis, both storms are in the transitional water-depth regimes since 0.5 < *k*_*p*_*d* < 2.5, as seen in the right panel of Fig. (2). Further, characteristics of both storms vary considerably over their durations of 70 hours, approximately. As a consequence, we propose novel probability models appropriate to non-stationary processes so as to be able to analyze the surface-elevation time series gathered during the two storms.

## Results

This section is structured as follows. First, we discuss the characteristics of the sea states generated by the two storms as they pass by the west coast of Ireland. The descriptions of various wavefield characteristics, associated principal statistical parameters and probability models employed in the analyses are described in the Methods section. Subsequently, we present the analysis of extreme waves. In particular, in order to be able to predict rationally the occurrence of extreme waves in a time-dependent storm, we first present the theoretical formulation of a non-stationary model for describing the sequences of sea states in a storm. On this basis, an optimal sea state duration is determined based on the rationale that variation of key statistics is minimal between two consecutive sea-state sequences. We then explore the occurrence frequency of rogue waves observed at a fixed point at sea. The largest waves observed at the peak of the storms and their characteristics are then compared to those of the Draupner and Andrea rogue waves, observed at different oil platforms in the North Sea in 1995 and 2007, respectively, the Killard rogue wave observed off the coast of Ireland in 2015^1^ and the simulated El Faro rogue wave^2^. The metocean parameters of the six sea states are summarized in Table 1.

**Table 1:** Wave parameters and various statistics of Doris and Feb 2015 at the storm peak in comparison to the El Faro^2^, Andrea, Draupner and Killard rogue sea states^1^.

	Doris	Feb 2015	El Faro	Andrea	Draupner	Killard
Significant wave height *H*_*s*_ [m]	6.4	12.6	9.0	10.0	11.2	11.4
Dominant wave period *T*_*p*_ [s]	10.0	14.1	10.2	14.3	15.0	17.2
Mean zero-crossing wave period *T*_0_ [s]	8.8	13.3	9.2	11.6	12.1	14.0
Mean wavelength *L*_0_ [m]	120	216	131	209	219	268
Depth *d* [m], *k*_0_*d* with *k*_0_ = 2*π*/*L*_0_	45, 2.35	37, 1.08	4700, 225	74, 2.23	70, 2.01	39, 0.91
Spectral bandwidth *ν*	0.46	0.51	0.49	0.35	0.36	0.37
Angular spreading *σ*_*θ*_ [*rad*]	0.90	$\tilde{{\rm{1}}}$.07	0.79	0.37	0.39	0.34
Parameter *R* = *σ*_*θ*_^2^/2*ν*^2^ ^37^	1.90	$\tilde{{\rm{2}}}$.2	1.34	0.56	0.59	0.42
Benjamin Feir Index *BFI*^38^	0.18	0.22	0.36	0.24	0.23	0.18
Narrow-band (NB) skewness *λ*_3,*NB*_^49^	0.190	0.221	0.262	0.159	0.165	0.145
Observed skewness *λ*_3_	0.144	0.441	0.162	0.141	0.146	0.142
Maximum NB dynamic excess kurtosis *λ*_40,*max*_^*d*^ ^39^	−10^−3^	−10^−1^	10^−3^	2.3 · 10^−3^	2.1 · 10^−3^	2.7 · 10^−4^
NB bound excess kurtosis *λ*_40,*NB*_^*d*^ ^41^	0.094	0.229	0.049	0.065	0.074	0.076
Observed excess kurtosis *λ*_40_	0.098	0.263	0.042	0.041	0.032	−0.011
Actual maximum crest height *h*/*H*_*s*_	1.11	1.23	1.68	1.63	1.55	1.44
Actual maximum crest-to-trough (wave) height *H*/*H*_*s*_	2.06	1.93	2.6	2.30	2.10	2.00

Note that the Killard rogue wave occurred on a water depth of 39 m, however the hincast input spectrum used by Fedele *et al*.^1^ could only be computed at an averaged water depth of 58 m. We refer to the Methods section for the definitions of wave parameters.

Our statistical analysis of large waves focused on the study of the time sequence of changing sea states during the two storms. An optimal sea state duration *T*_*sea*_ of 50 minutes for both storms was determined so as to minimize the degree of difference between waves of consecutive sea states. Drawing on Boccotti^9^, this is measured by the standard deviation of the random variable *V* = *σ*_2_/*σ*_1_ − 1, where *σ*_*j*_^2^ are the variances of two successive sea states in the storm sequence as shown in Fig. (2). Sampled values of *V* are obtained by dividing the non-stationary time series into *N* = *D*_*s*_/*T*_*sea*_ successive sea states of the same duration *T*_*sea*_ and variances *σ*_1_^2^,*σ*_2_^2^, ..., *σ*_*j*_^2^,*σ*_*j*_ _+ __1_^2^, ..., where *D*_*s*_ is the storm duration (70 hours). Then, *N* − 1 sampled values of *V* follow as *V*_*j*_ = *σ*_*j*_ _+_ _1_/*σ*_*j*_ − 1 for *j* = 1, ..., *N* − 1, from which the standard deviation of *V* can be estimated. Obviously, the mean of *V* tends to zero as *T*_*sea*_ approaches smaller values.

This process ensured that resulting statistics are robust to variations in *T*_*sea*_ up to ±20 min once the total population of surface elevations from each sea state in a storm sequence are normalized by the respective significant wave height.

### Metocean parameters

Both storms generated directional sea states in transitional water depths. This is clearly seen in the right panel of Fig. (2), displaying the hourly variations of the depth coefficient *k*_*p*_*d* during the two storms. Surface spectra observed were broadbanded so that *ω*_*p*_ ≈ 0.8*ω*_*m*_, while *k*_*p*_ ≈ 0.7*k*_*m*_.

**Figure 3:**
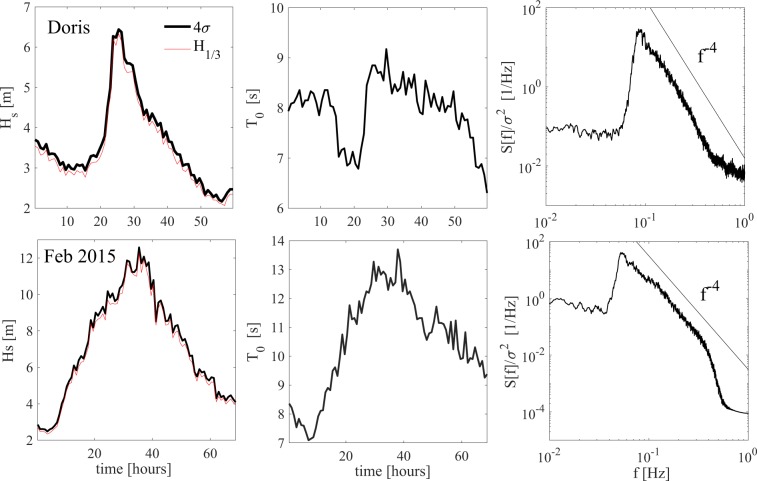
History of metocean parameters in (top panels) Doris and (bottom panels) Feb 2015 storms: (left) hourly variations of significant wave height *H*_*s*_ = 4*σ* compared to actually observed *H*_1/3_, (center) variation of mean zero-up-crossing period *T*_0_, and (right) surface spectra at peak stage of storms and their high-frequency saturation compared to a logarithmic *f*^−4^ type decay.

Metocean parameters of both storms and how these vary are shown below in the top and bottom panels of Fig. (3) respectively. In particular, the left panels of Fig. (3) depict the hourly variation of the significant wave height *H*_*s*_ = 4*σ*. For comparison, the variation of actual significant height *H*_1/3_ representing the mean of the highest 1/3 of wave heights observed is also shown in the same panels. It is seen that it is about 5% smaller than *H*_*s*_. The actual mean zero-up-crossing wave period *T*_0_ is also shown in the center panels of the same figure, whereas the right panels depict the wave spectra measured at the storm peaks. We observe that the high-frequency behavior in both cases is described by a logarithmic *f*^−4^ decay in conformity with Zakharov’s wave turbulence^20^.

**Figure 4:**
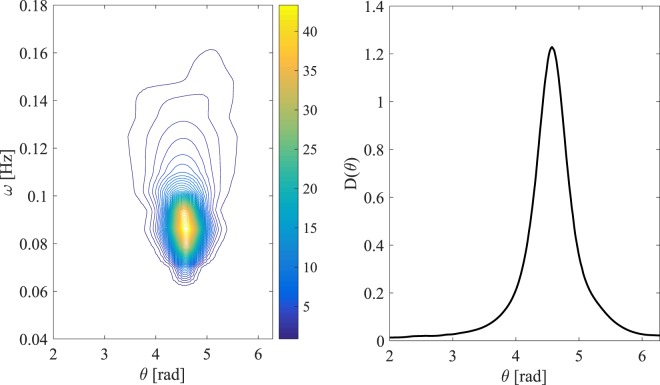
Doris: (left panel) Estimated normalized directional spectrum *S*_*d*_(*ω*,*θ*)/*σ*^2^ [*Hz*^−1^rad^−1^] at the storm peak using the Bayesian direct method (BDM) and (right panel) associated angular spreading function $D(\theta )={\int }_{0}^{\infty }{S}_{d}(\omega ,\theta ){\rm{d}}\omega /{\sigma }^{2}$.

The two states analyzed here do not present any characteristics typical of mixed or crossing seas such as swell waves overlapping with wind seas because the frequency spectra *S*(*f*) displays a unimodal structure, as depicted in the right panels of Fig. (3). In particular, an examination of the directional spectrum *S*_*d*_(*ω*, *θ*)/*σ*^2^ estimated using the Bayesian direct method (BDM) at the peak of Doris storm and shown in the left panel of Fig. (4) clearly displays a unimodal broad-banded wind-wave field, also confirmed by the attendant unimodal directional spreading function *D*(*θ*) in the right panel of the same figure.

**Figure 5:**
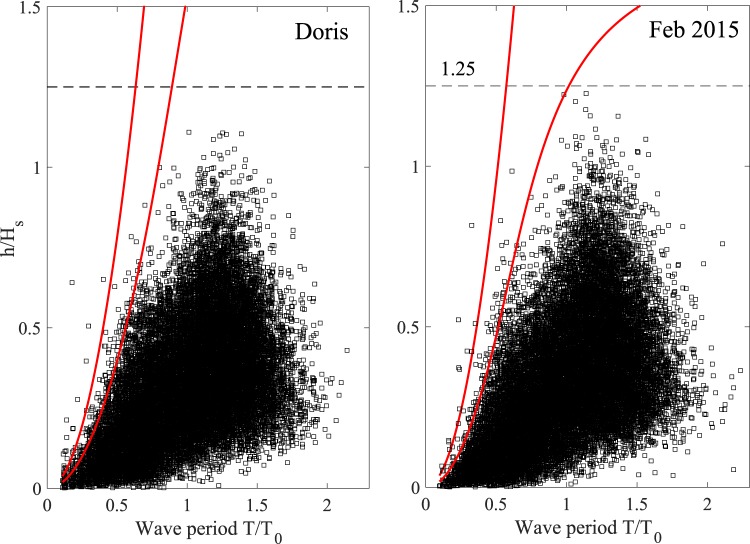
Scatter diagrams of crest heights *h*/*H*_*s*_ versus wave periods *T*/*T*_0_ observed in Doris and Feb 2015 storms compared with Miche-Stokes limits for the weakest (upper red curves) and the most intense (lower red curves) sea states, respectively.

Figure (5) displays the scatter diagrams of crest heights *h*/*H*_*s*_ versus corresponding wave periods *T*/*T*_0_ for both storms. Large crest heights (and similarly wave heights, not reported here) do not violate the Miche-Stokes limits. These are depicted in the same figure by two bold red lines representing the Miche-Stokes limits for the most intense and weakest sea states of the storms. In seas generated by intense storms, nonlinear wave dispersion is effective in limiting wave growth as a precursor to breaking^21–23^. Thus, the onset of wave-breaking can occur well below the Miche-Stokes upper limit^22,24–27^.

**Figure 6:**
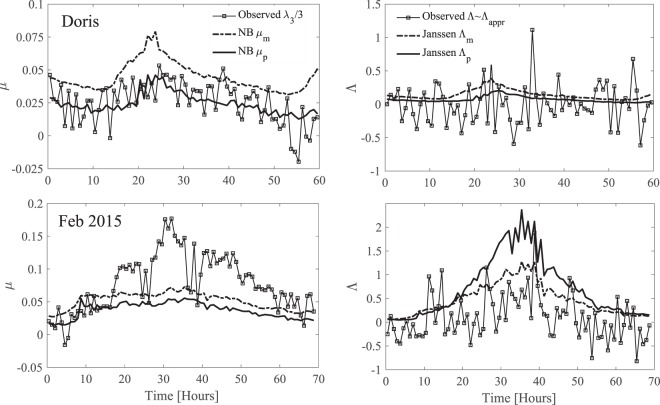
Histories of statistical parameters in (top panels) Doris and (bottom panels) Feb 2015 storms: (left) hourly variation of observed values of *μ* = *λ*_3_/3^6^ compared to theoretical NB steepnesses^3,4^ *μ*_*m*_ and *μ*_*p*_; (right) hourly variation of observed Λ and Λ_*appr*_ = 8*λ*_40_/3 implied by NB theory^41^ compared to NB estimates Λ_*m*_ and Λ_*p*_. Note that Λ is practically the same as Λ_appr_. Subscripts *m* and *p* refer to definitions of parameters based on the mean and dominant wavenumbers *k*_*m*_ and *k*_*p*_, respectively.

In Table 1 we compare the metocean parameters observed during the peak states of the two storms with those of the El Faro, Draupner, Andrea and Killard rogue sea states^1^. Clearly, all six sea states have similar metocean characteristics. Killard, Doris and Feb 2015 are in shallower waters and the last two have a greater steepness than the other four sea states. Indeed, the observed values of skewness *λ*_3_ and excess kurtosis *λ*_40_ are larger than those observed in the other four cases (see also Fig. (6)). This suggests that the largest waves observed were near the onset of incipient breaking or already breaking, thus lessening the likelihood of occurrence of larger rogue events^21,22,28^.

### Modulational instability in intermediate or transitional water depths

In the Feb 2015 storm, the depth coefficient *k*_*p*_*d*, depicted in the right panel of Fig. (2), was below the critical depth threshold 1.363 whereas not so in Doris. Above the threshold, plane waves are modulationally unstable^29^ in the one-dimensional (1-D) wave dynamics described by the Nonlinear Schrödinger (NLS) equation^30,31^. However, the wave fields analyzed here are directional sea states, and according to the 2-D hyperbolic NLS equation, plane waves are modulationally unstable even at depths below that critical value, if they are perturbed by appropriate oblique disturbances^29,32–34^. Nevertheless, it is also recognized that instabilities ensuing from such disturbances are not likely to occur for values of *k*_*p*_*d* < 0.5^32^. So, it is plausible that rogue waves could be generated by modulational instability, as in unidirectional seas^35,36^ during both of the storms analyzed here. The kurtosis statistics is often used as an indicator if any rogue waves are present in a sea state. In sea states where third-order nonlinearities are significant, excess kurtosis *λ*_40_ = *λ*_40_^*d*^ + *λ*_40_^*b*^ comprises a dynamic component^37–40^ *λ*_40_^*d*^ due to nonlinear quasi-resonant wave-wave interactions and a Stokes bound harmonic contribution^40,41^ *λ*_40_^*b*^, given in the Methods section. As for the dynamic component, drawing on Janssen^40^, Fedele’s^39^ one-fold integral formulation is extended to narrowband (NB) waves in intermediate waters as

1 $${\lambda }_{40}^{d}=6{\alpha }_{S}BF{I}^{2}\,{\rm{Im}}{\int }_{0}^{{\nu }^{2}{\omega }_{p}\,t}\frac{1}{\sqrt{1-2i\alpha +3{\alpha }^{2}}\sqrt{1+2i{R}_{S}\alpha +3{R}_{S}^{2}{\alpha }^{2}}}d\alpha \mathrm{.}$$

In the preceding expression, $BFI=\sqrt{2}{k}_{p}\sigma /\nu$ defines the Benjamin-Feir index in deep water at the spectral peak, *ν* the spectral bandwidth, $i=\sqrt{-1}$ and Im(*x*) denotes the imaginary part of *x*, *ω*_*p*_ and *k*_*p*_ the dominant spectral frequency and wavenumber. Depth effects on wave directionality, measured by *R*, are represented by *R*_*S*_ = *β*_*S*_*R* by way of the factor *β*_*S*_, and *α*_*S*_ is the depth factor. The latter two depend on the dimensionless depth *k*_*p*_*d* (see Methods section). In the deep-water limit, both *α*_*S*_ and *β*_*S*_ become 1. The maximum of dynamic excess kurtosis is well approximated by^39^

2 $${\lambda }_{\mathrm{40,}\,{\rm{\max }}}^{d}({R}_{S})=3{\alpha }_{S}BF{I}^{2}\frac{b}{{\mathrm{(2}\pi )}^{2}}\frac{1-{R}_{S}}{{R}_{S}+b{R}_{0}},\,0\le {R}_{S}\le \mathrm{1,}$$

and

3 $${\lambda }_{\mathrm{40,}\,{\rm{\max }}}^{d}({R}_{S})=-\frac{1}{{R}_{S}}{\lambda }_{\mathrm{40,}\,{\rm{\max }}}^{d}(\frac{1}{{R}_{S}}),\,{R}_{S} > \mathrm{1,}$$

where ${R}_{0}=3\sqrt{3}/4{\pi }^{3}$ (which corrects a misprint in^39^) and *b* = 2.48.

In the focusing regime (0 < *R*_*S*_ < 1 and *α*_*S*_ > 0) the dynamic excess kurtosis of an initially homogeneous Gaussian wave field grows, reaches a maximum at the intrinsic time scale ${\tau }_{c}={\nu }^{2}{\omega }_{p}{t}_{c}=1/\sqrt{3{R}_{S}}$ and then monotonously decreases and eventually vanishes over longer times. Such a regime is typical of unidirectional narrowband waves on water depths *k*_*p*_*d* < 1.363. Indeed, in 1-D waves modulational instability disappears below that critical threshold and *α*_*S*_ < 0. As a result, wave dynamics becomes of defocusing type and excess kurtosis is negative. In particular, it reaches a minimum at *t*_*c*_ and then tends to zero over larger times. The kurtosis formulation in Eq. (1) extends Fedele’s^39^ stochastic approach to NB waves at intermediate water depths, and it indicates that in directional seas such as those analyzed in this study, modulation instability can also occur at depths below the critical threshold 1.363 for *α*_*S*_ > 0 (see also Alber^42^). Further, for waves propagating over a broad range of directions in the open sea, Fedele *et al*.^1^ show that such instabilities are ineffective in triggering rogue waves as excess kurtosis becomes negative, provided that *R*_*S*_ > 1. A rogue wave regime is a more likely occurrence only if the surface spectrum is sufficiently narrow-banded (*R*_*S*_ < 1) as well as characterized by a relatively large positive excess kurtosis.

**Figure 7:**
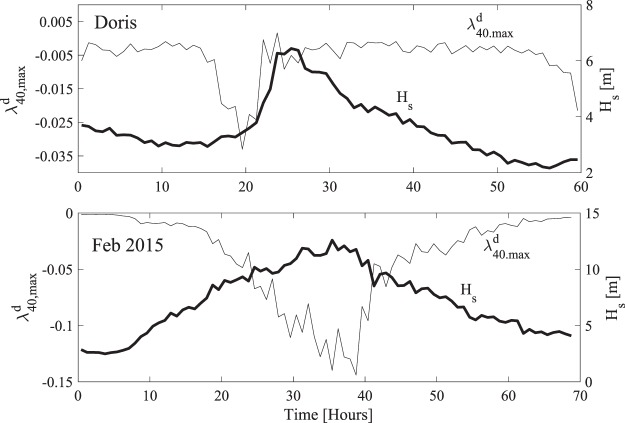
Variations of hourly estimates in (top panel) Doris and (bottom panel) Feb 2015 storms of the maximum of dynamic excess kurtosis *λ*_40_^*d*^ (thin line) and significant wave height *H*_*s*_ (thick line).

Both storms analyzed here are in transitional water depths and prone to potential rogue occurrences induced by modulational instability since *α*_*S*_ > 0. However, all their sea states are directionally spread and characterized with mostly negative excess kurtosis since *R*_*S*_ > 1. This can clearly be seen in Fig. (7). Thus, the potential recurrence or focusing of large waves as observed in unidirectional seas is largely attenuated or suppressed^1,2,7,39^. Indeed, our statistical analysis indicates that the effects of third-order resonance or modulational instabilities are negligible, and that second-order bound nonlinearities are the dominant factor in shaping the large waves observed. We also point out that the NB predictions based on the mean wavenumber *k*_*m*_ yield similar negligible estimates of the dynamic excess kurtosis.

In summary, our analysis indicates that fourth order cumulants are essentially trivial to begin with, implying that the analyzed sea states are ordinary: nothing specially rogue about them. The present analyses of the two storm wave datasets discussed in the following section confirm all this and show that there is hardly anything beyond second-order nonlinearities to explain their statistics.

### Nonlinear wave statistics

The relative importance of nonlinearities in a sea state can be assessed by way of various integral statistics. These include the observed values of the wave steepness *μ* = *λ*_3_/3 ^6^ and the coefficient Λ = *λ*_40_ + 2*λ*_22_ + *λ*_04_ ^4^, where *λ*_3_ is skewness and *λ*_*ij*_ are fourth-order cumulants of the zero-mean surface elevation *η*(*t*) and its Hilbert transform. In particular, *λ*_40_ is the excess kurtosis of surface elevations. Skewness is a measure of vertical asymmetry, and it describes the effects of second-order bound nonlinearities on the geometry and statistics of the sea surface with higher sharper crests and shallower more rounded troughs^3,4,6^. Excess kurtosis indicates whether the tail of the distribution of surface elevations is heavier or lighter relative to a Gaussian distribution. It comprises a dynamic component *λ*_40_^*d*^ measuring third-order quasi-resonant wave-wave interactions and a bound contribution *λ*_40_^*b*^ induced by both second- and third-order bound nonlinearities^3–6,37,43^.

In describing wave statistics, the theoretical NB predictions based on the mean wavenumber *k*_*m*_, rather than *k*_*p*_, tend to yield more favorable comparisons with deep-water observations or theories^6,44^. Definitions based on *k*_*p*_ lead to predictions that noticeably underestimate the observed and/or theoretical statistics of broadband waves since *k*_*p*_ < *k*_*m*_^44^. Nonetheless, the sea states analyzed here are in intermediate water depths and characterized with broad-banded spectra both in frequency and direction. Describing the statistics in such cases based on either *k*_*m*_ or *k*_*p*_ is neither very reliable nor realistic.

In Table 1 we compare the statistical parameters of the most intense sea states of Doris and Feb 2015, and also the Draupner, Andrea and Killard rogue sea states^1,2^. The maximum dynamic excess kurtosis is negative and negligible. Thus, third-order quasi-resonant interactions, including NLS-type modulational instabilities, should not play any significant role in the formation of large waves in comparison to bound nonlinearities^1,39^ especially as the wave spectrum broadens^21^ in agreement with oceanic observations available so far^4,45,46^. The values of excess kurtosis *λ*_40_ and Λ are mostly due to bound nonlinearities^7,47,48^.

In the top panels of Fig. (6), we compare the hourly variations of (left) the observed values of *μ* = *λ*_3_/3 ^6^ versus the theoretical NB approximations^3,49^ *μ*_*m*_ and *μ*_*p*_ based on the mean and dominant wavenumbers *k*_*m*_ and *k*_*p*_, respectively, and (right) the observed fourth–order Λ coefficient, its approximation Λ_appr_ and the NB predictions^37,41^ Λ_*m*_ and Λ_*p*_ based on *k*_*m*_ and *k*_*p*_ for Doris. The same comparisons are also reported in the bottom panels for Feb 2015. Clearly, the two NB predictions *μ*_*m*_ overestimate and *μ*_*p*_ slightly underestimate the observed values of *μ* for Doris. In contrast, both NB predictions underestimate the observed steepness for Feb 2015. Similarly, the actual Λ is mostly overestimated by both the NB estimates. Moreover, Λ is practically the same as Λ_appr_ (see Methods section). In the final analysis, the sea states analyzed here are characterized by broadband spectra both in frequency and direction and the NB assumption is unrealistic. Thus, hereafter we use the observed values of *μ* and Λ to evaluate wave statistics.

### Occurrence frequency of extreme waves in storms

We now describe a novel approach for the statistics of extreme waves encountered by an observer at a fixed point of the ocean surface during a storm of duration *D*_*s*_. Drawing on^9,50–52^, the storm is modeled as a non-stationary continuous sequence of sea states of duration *dt*, and *dt*/*T*_0_(*t*) is the number of waves in the sea state, where *T*_0_(*t*) is the time-changing mean zero-crossing wave period. Consider now a wave of the storm and define the probability *P*_*ns*_(*ξ*) that its crest height *C* exceeds the threshold *h* = *ξH*_*s*_ as observed at a fixed location where *H*_*s*_ = 4*σ*. Equivalently, this is the probability of randomly picking a wave crest whose height *C* exceeds the threshold *ξ* = *h*/*H*_*s*_ from the non-stationary time series observed at a fixed point of the storm. Then,

4 $${P}_{ns}(\xi )=\frac{{\int }_{0}^{{D}_{s}}P(\xi ,t)\frac{1}{{T}_{0}(t)}dt}{{\int }_{0}^{{D}_{s}}\frac{1}{{T}_{0}(t)}dt},$$

where *P*(*ξ*, *t*) = Pr[*C* > *ξH*_*s*_(*t*)] stands for the probability that a wave crest height *C* exceeds the threshold *ξH*_*s*_(*t*) in the sea state occurring in the time interval [*t*, *t* + *dt*]. This probability depends on wave parameters around time *t*. The definition of *P*_*ns*_ is consistent with the way wave crests are sampled from non-stationary wave measurements during storms. In particular, a storm is partitioned into a finite sequence of *N*_*s*_ sea states of duration *T*_*sea*_ = *D*_*s*_/*N*_*s*_. In each sea state *S*_*j*_ centered at *t* = *t*_*j*_, waves are sampled and their crest elevations (*h*) are normalized with the local significant wave height as *h*/*H*_*s*_(*t*_*j*_) and put all in the same population . Then, the empirical exceedance probability *P*_*ns*_(*ξ*) is estimated as the ratio of the number of waves that exceed *ξ* to the total number of waves in the population. This is consistent with the way Eq. (4) is formulated. Indeed, ${N}_{w}(t,dt)=EX(t)dt=\frac{1}{{T}_{0}(t)}dt$ is the expected number of waves during a sea state in [*t*, *t* + *dt*] and *P*(*ξ*, *t*)*EX*(*t*)*dt* is the number of waves whose crest heights exceed the threshold *h* = *ξH*_*s*_(*t*) in the same sea state. Then, *P*_*ns*_ in Eq. (4) follows by cumulating (integrating over time) the number of waves of all the sea states whose crest heights exceed *h*. In practice, the non-stationary *P*_*ns*_ is estimated from data as the weighted average

5 $${P}_{ns}(\xi )=\frac{{\sum }_{j=1}^{{N}_{s}}{\rm{\Pr }}[C > \xi {H}_{s}({t}_{j})]{N}_{w}({t}_{j})}{{\sum }_{j\mathrm{=1}}^{{N}_{s}}{N}_{w}({t}_{j})},$$

where *N*_*w*_(*t*_*j*_) is the number of waves sampled in the sea state *S*_*j*_. Equation (4) also implies that the threshold *ξH*_*s*_ is exceeded on average once every *N*_*h*_(*ξ*) = 1/*P*_*ns*_(*ξ*) waves. Thus, *N*_*h*_ can be interpreted as the conditional return period of a wave whose crest height exceeds *ξ*. For weakly nonlinear seas, the probability *P*(*ξ*, *t*) is hereafter described by the third-order Tayfun-Fedele^4^ (TF), second-order Tayfun^3^ (T), Forristall^8^ (F) and the Rayleigh (R) distributions (see Methods section). Note that we are now able to estimate the probability *P*_*ns*_(*ξ*) that a wave of the storm has a crest height *C* larger than *ξ* = *h*/*H*_*s*_. However, we still need to find in what sea state (of significant wave height *H*_*s*_) such a wave most likely occurs.

To do so, we draw on^9,51,52^ and express the probability density function (pdf) describing the time at which a wave crest *C* exceeds a specified or given level *h* in the interval [*t*, *t* + *dt*] as

6 $$p(t|h)=\frac{P[\xi =h/{H}_{s}(t)]\frac{1}{{T}_{0}(t)}}{{\int }_{0}^{{D}_{s}}P[\xi =h/{H}_{s}(\tau ),\tau ]\frac{1}{{T}_{0}(\tau )}d\tau }.$$

**Figure 8:**
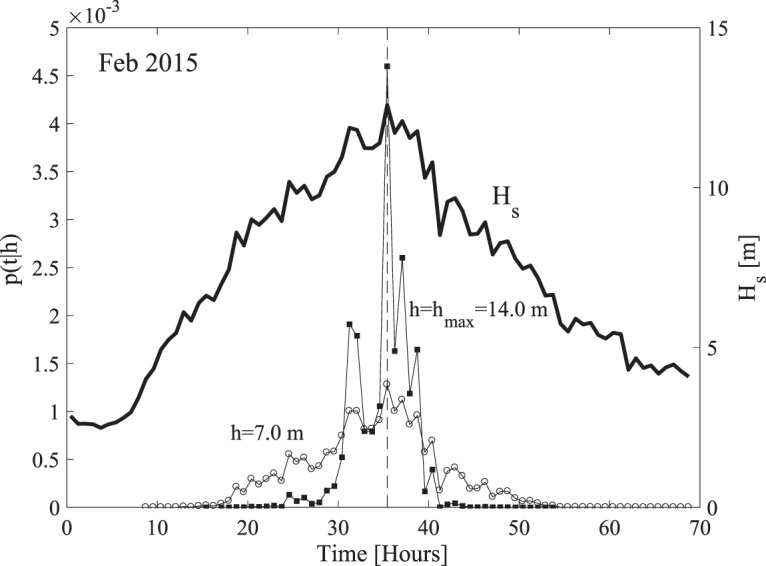
Probability density function *p*(*t*|*h*) [1/min] of a wave whose crest elevation exceeds the maximum observed height *h* = *h*_*max*_ = 1.23*H*_*s*_ = 14 m in the Feb 2015 storm. Hourly variations (bold line) of significant wave height *H*_*s*_ are also shown. The vertical dashed line indicates the sea state in which the largest crest was observed. For comparison, the pdf for *h* = *h*_*max*_/2 = 7 m is also shown.

The preceding pdf is estimated from data as

7 $$p({t}_{j}|h)=\frac{P[\xi =h/{H}_{s}({t}_{j})]{N}_{w}({t}_{j})/{T}_{sea}}{{\sum }_{k=1}^{{N}_{s}}P[\xi =h/{H}_{s}({t}_{k})]{N}_{w}({t}_{k})},$$

where *T*_*sea*_ is the sea state duration. As an example, consider the Feb 2015 storm. The largest wave with also the largest crest height of *h* = *h*_*max*_ = 1.23*H*_*s*_ = 14 m (see Table 1) is observed in the sea state at the storm peak (*H*_*s*_ = 12.6m). The pdf *p*(*t*|*h*) estimated for that crest amplitude, and shown in Fig. 8, is very narrow and concentrated around its absolute maximum coincident with the storm peak in agreement with what is expected intuitively. Instead, waves whose crest height exceeds the smaller threshold *h* = *h*_*max*_/2 = 7 m have a pdf that still has its maximum at the storm peak, but it is broader indicating that crest heights exceeding 7 m likely occur also before or after the storm peak.

Similarly, the nonstationary occurrence frequency of a wave of the storm whose crest-to-trough wave height exceeds the threshold *H* as well as the pdf *p*(*t*|*H*) of the sea state in which such waves occur can be both described by the same Eqs ((4), (6)) by simply replacing *P*(*h*) with the exceedance probability *P*(*H*) appropriate for wave heights of stationary seas. This is hereafter described by the generalized Boccotti (B), Tayfun (T) and linear Rayleigh (R) distributions (see Methods section).

Finally, the second-order nonstationary pdf *p*_*ns*_(*z* = *η*/*σ*) of wave surface elevations *η* for storms is defined as

8 $${p}_{ns}(\eta /\sigma )=\frac{{\int }_{0}^{{D}_{s}}\,p(\eta /\sigma (t))\frac{1}{{T}_{0}(t)}dt}{{\int }_{0}^{{D}_{s}}\,\frac{1}{{T}_{0}(t)}dt},$$

where *p*(*z* = *η*/*σ*) is the Tayfun approximation^3,44^ for nonlinear stationary sea states, described by

9 $$p(z)=\frac{1}{\sqrt{2\pi }}\frac{\exp (-\frac{{F}^{2}}{2{\mu }^{2}})}{F+1},\,z > -\frac{1+{\mu }^{2}}{2\mu },$$

where $F=\sqrt{1+2\mu z+{\mu }^{2}}-1$ and *μ* = *λ*_3_/3, valid for the observed values of skewness coefficient *λ*_3_ < 0.6, approximately.

Note that the probability structure of storm-wave characteristics depends upon the time history of wave parameters, say *α*(*t*), such as significant wave height, skewness and excess kurtosis. The analyses of the data sets here indicate that the non-stationary distributions are well approximated by the corresponding stationary models of an equivalent sea state with duration equal to that of the actual storm (as if *H*_*s*_ does not vary in time) evaluated using the weighted average parameters

10 $$\tilde{\alpha }=\frac{{\int }_{0}^{{D}_{s}}\,\alpha (t)\frac{1}{{T}_{0}(z)}dt}{{\int }_{0}^{{D}_{s}}\frac{1}{{T}_{0}(z)}dt}\approx \frac{{\sum }_{j\mathrm{=1}}^{{N}_{s}}\alpha ({t}_{j}){N}_{w}({t}_{j})}{{\sum }_{j\mathrm{=1}}^{{N}_{s}}{N}_{w}({t}_{j})}\mathrm{.}$$

This follows from modeling the actual storm as if it had an ‘equivalent’ rectangular shape and assuming that the average parameters of the Tayfun and Boccotti models are the same in both the actual and equivalent storms. However, such approximations do not have general validity, and they may not work for other models or data sets. Thus, hereafer we will only consider the non-stationary models based on (4).

**Figure 9:**
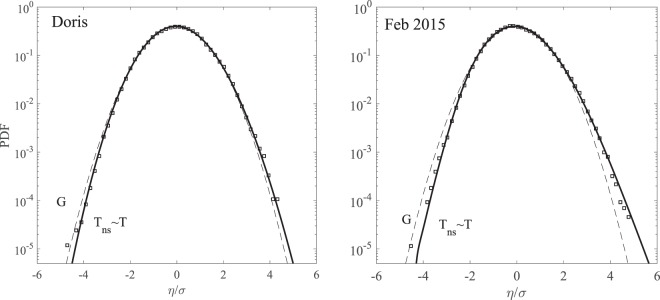
Probability density function of scaled surface wave elevations *η*/*σ* in (left) Doris and (right) Feb 2015 storms. The empirical distributions (□) derived from the total wave population are compared with the non-stationary second-order Tayfun (T) distribution for storms. Dashed curves (G) describe the probability density for a standard unit Gaussian variable.

The empirical distributions of surface wave elevations for both storms Doris and Feb 2015 are shown in Fig. (9). These are for the most part well described by the non-stationary Tayfun pdf *p*_*ns*_, which is practically the same as the stationary approximation estimated using the weighted average steepness *μ* based on Eq. (10). This indicates the dominance of second-order nonlinearities in shaping the sea surface, especially for the more intense Feb 2015 storm.

**Figure 10:**
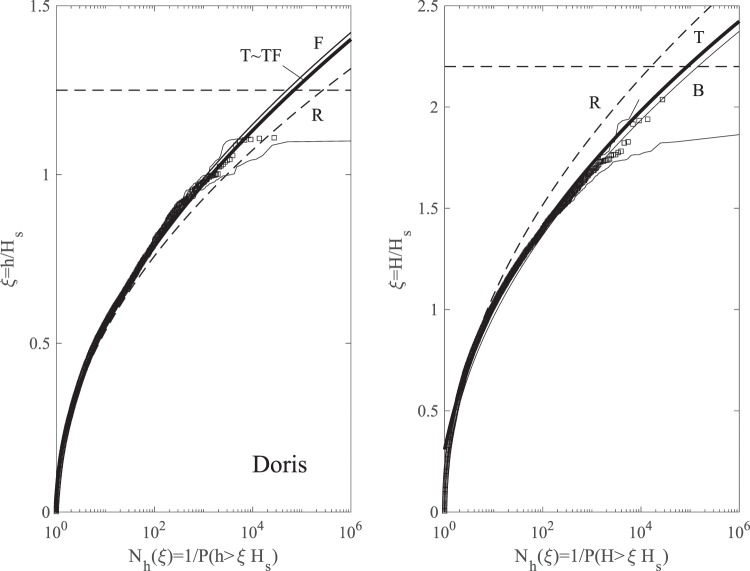
Doris: (left) crest heights *h*/*H*_*s*_ and (right) crest-to-trough wave heights *H*/*H*_*s*_ versus number of waves *N*_*h*_(*ξ*). Empirical distributions (□) of total population of waves observed in comparison with non-stationary models (T = Tayfun, TF = Tayfun-Fedele, F = Forristall, B = generalized Boccotti and R = Rayleigh distributions). (Light gray lines) approximate confidence bands on observational estimates and (horizontal dashed lines) rogue thresholds^58^ for crest (1.25*H*_*s*_) and wave heights 2.2*H*_*s*_, respectively.

**Figure 11:**
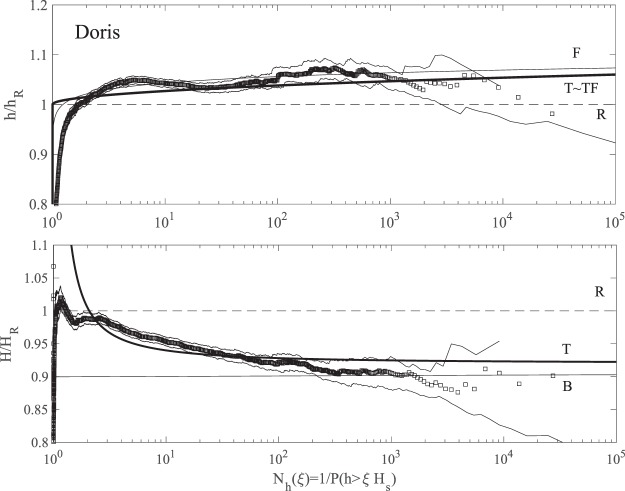
Doris: (top) crest heights *h*/*h*_*R*_ and (bottom) crest-to-trough wave heights *H*/*H*_*R*_ versus number of waves *N*_*h*_(*ξ*). Empirical distributions (□) of total population of waves observed in comparison with non-stationary models (T = Tayfun, TF = Tayfun-Fedele, F = Forristall, B = generalized Boccotti and R = Rayleigh distributions). (Light gray lines) approximate confidence bands on observational estimates. Amplitude *h*_*R*_ (*H*_*R*_) refers to Rayleigh-distributed crest (crest-to-trough) heights exceeded with probability *P*(*ξ*) in Gaussian seas.

Figure (10) summarizes the wave statistics for Doris. In particular, the left panel of the figure depicts the empirical distribution for crest heights *h*/*H*_*s*_ observed in the total wave population plotted versus the number of waves *N*_*h*_(*ξ*). This is compared against the theoretical predictions of the nonstationary second-order Tayfun (T), third-order Tayfun-Fedele (TF), Forristall (F) as well as the Rayleigh (R) distributions. Although the associated confidence bands on the empirical probabilities noticeably widen over the large waves, the theoretical predictions nonetheless still lie mostly within the same confidence bands. Note that TF is practically the same as T and F as an indication that second-order effects are dominant, whereas the linear R model underestimates the return periods. Similarly, the right panel of the same figure shows the empirical distribution for crest-to-trough wave heights *H*/*H*_*s*_. The observed statistics is well described by both the non-stationary generalized Boccotti (B) and Tayfun (T) models. The small differences among the various models are magnified in Fig. (11), which shows the plots of the normalized crest height *h*/*h*_*R*_ and wave height *H*/*H*_*R*_ versus the number of waves *N*_*h*_. Here, *h*_*R*_ and *H*_*R*_ are the crest and crest-to-trough-thresholds exceeded with probability 1/*N*_*h*_ in a Gaussian sea in accord with the Rayleigh law.

**Figure 12:**
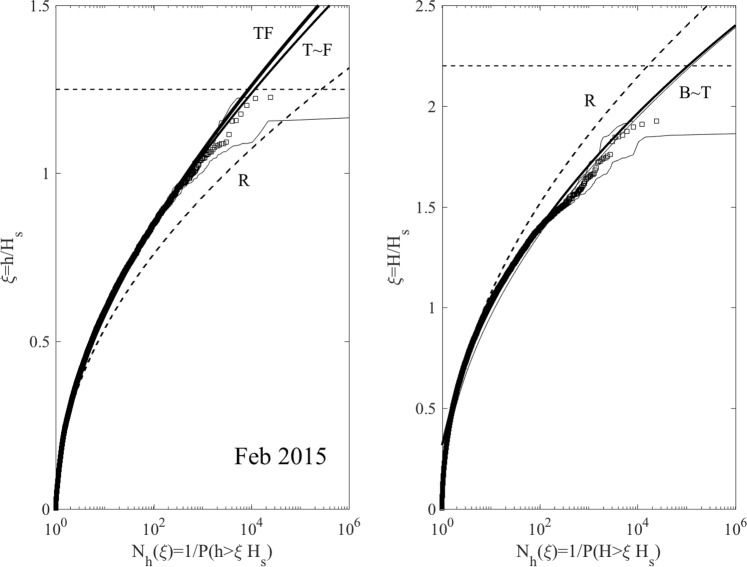
Feb 2015: (left) crest heights *h*/*H*_*s*_ and (right) crest-to-trough wave heights *H*/*H*_*s*_ versus number of waves *N*_*h*_(*ξ*). Empirical distributions (□) of total population of waves observed in comparison with non-stationary models (T = Tayfun, TF = Tayfun-Fedele, F = Forristall, B = generalized Boccotti and R = Rayleigh distributions). (Light gray lines) approximate confidence bands on observational estimates and (horizontal dashed lines) rogue thresholds^58^ for crest (1.25*H*_*s*_) and wave heights 2.2*H*_*s*_, respectively.

**Figure 13:**
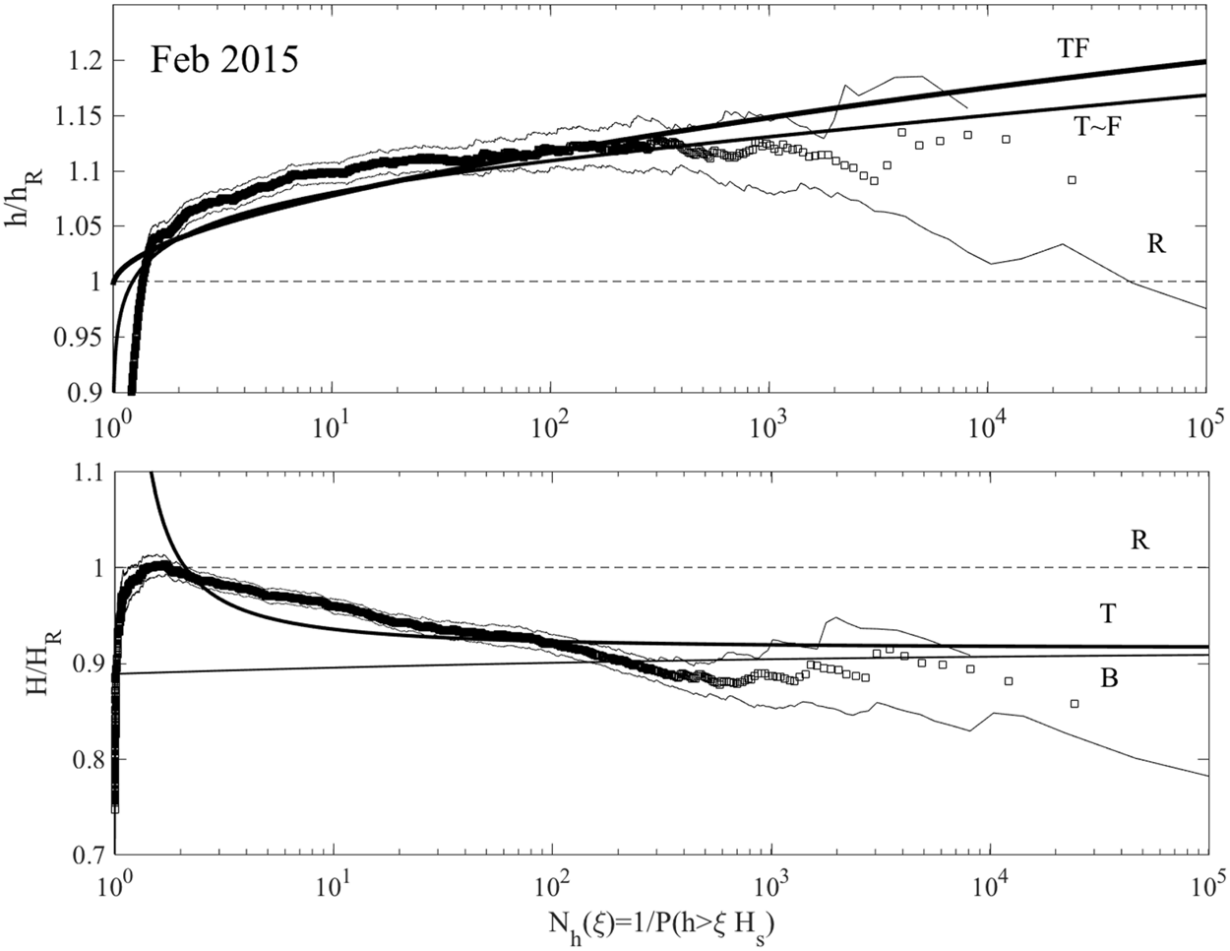
Feb 2015: (top) crest heights *h*/*h*_*R*_ and (bottom) crest-to-trough wave heights *H*/*H*_*R*_ versus number of waves *N*_*h*_(*ξ*). Empirical distributions (□) of total population of waves observed in comparison with non-stationary models (T = Tayfun, TF = Tayfun-Fedele, F = Forristall, B = generalized Boccotti and R = Rayleigh distributions). (Light gray lines) approximate confidence bands on observational estimates. Amplitude *h*_*R*_ (*H*_*R*_) refers to Rayleigh-distributed crest (crest-to-trough) heights exceeded with probability *P*(*ξ*) in Gaussian seas.

Similar conclusions also hold for the wave statistics for the Feb 2015 storm, summarized in Figs (12) and (13). As regard to crests, TF slightly exceeds T, again as an indication that second-order effects are dominant.

**Figure 14:**
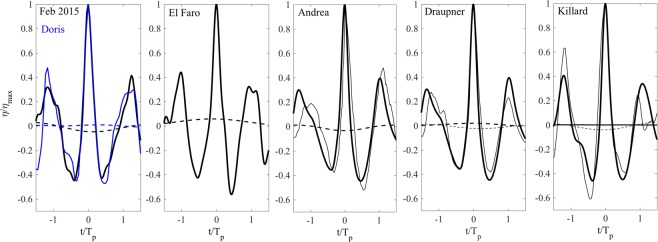
Observed wave profiles *η*/*η*_*max*_ (thick solid) and mean sea levels (MSL) (thick dashed) versus *t*/*T*_*p*_ for (first panel from the left) Doris and Feb 2015, and (following panels from left to right) El Faro, Andrea, Draupner and Killard simulated waves (thin solid) and MSL (thick dashed). Actual measurements (thick solid) and MSLs (thick solid) are also shown for Andrea, Draupner and Killard. Note that the Killard MSL is insignificant and the Andrea MSL is not available. *η*_*max*_ is the maximum crest height and *T*_*p*_ is the dominant wave period (see Table 1 and Methods section for definitions).

The wave profiles *η* with the largest wave crest height observed during Doris and Feb 2015 are shown in the left panel of Fig. (14). In the other panels, we display the El Faro, Draupner, Andrea and Killard rogue wave profiles for comparison^1,2^. In the same figure, the mean sea level (MSL) below the crests is also shown. The estimation of the MSL follows by low-pass filtering the time series of zero-mean surface elevations with a frequency cutoff ${f}_{c} \sim {f}_{p}/2$, where *f*_*p*_ is the frequency at the spectral peak^53^.

All six wave profiles are similar, suggesting a common generation mechanism for rogue events. In particular, all cases have sharper crests and rounded troughs and they do not display any secondary maxima or minima. They appear more regular and behave as narrow-banded waves do^45^. In essence, this means that the temporal profile of a relatively large wave observed over a complete phase cycle of 2*π* displays a single dominant crest or a ‘global’ maximum with no local maxima or minima. In other words, the wave phase monotonously increases over the cycle without any reversals associated with local minima and maxima^45^. These characteristics typical of truly narrow-band waves in every wave cycle are similarly observed but locally in the largest group of waves in a wind-wave field although they are not in the least described by a narrow-banded spectrum. In narrow-band waves, the constructive interference is the primary mechanism for the generation of large displacements in the underlying first-order linear field. The second-order corrections are phase-locked to the linear field such that they always tend to enhance wave crests and flatten the troughs, leading to the basic vertical asymmetry observed in oceanic waves. The process is similar for relatively large waves in a wind-wave field^45^.

Further, Doris and Feb2015 both display the characteristics of a dominant wind wave field and show no evident characteristics typical of mixed or crossing seas such as swell overlapping with local wind waves (see e.g. Fig. 4). That may explain the minor set-down observed below the largest waves observed. On the contrary, a set-up below the simulated El Faro and actual Draupner rogue waves is observed, most likely due to the multidirectionality of the respective sea states^2^. Indeed, recent studies showed that Draupner occurred in mixed seas consisting of swell waves propagating at approximately 80 degrees to wind seas^54–56^. Instead, the El Faro sea state showed a very broad directional spreading of energy typical of strong hurricane conditions. The multi-directionality of the two sea states may explain the set-up observed under the large wave^53^ instead of the second-order set-down normally expected^57^.

## Discussion

There is at present no consistent crest-height or wave-height model that works effectively at shallow water depths where *kd* < *π*/10 and recent comparisons^15^ simply serve to demonstrate this. The wave regime in such shallow waters can only be described by stochastic formulations of highly nonlinear shallow-water equations. Second-order theories or approximations tend to become ineffective at such depths. Our work does not overlap with or extend to such water depths where the second-order theory breaks down. Indeed, we have shown that the theoretical Tayfun^3,6^ and Boccotti^9,10^ models for crest and wave heights, largely applied to and validated for deep-water waves^4,10^ and more recently for mixed/crossing seas^11–13^, describe waves reasonably well in intermediate shallow waters also (*π*/10 < *kd* < *π*). So does the second order Forristall model^8^.

In particular, we have analyzed actual wave data from ADCP measurements gathered during the passages of two major storms nearshore off Killard Point at the intermediate water depth of approximately 37 m (*k*_*p*_*d* = 0.6–2.5) in 2015 and off the Aran Islands at 45 m depth (*k*_*p*_*d* = 1.36–2.2) in 2017 (see Fig. 1). The observed sea states at the storm peak present the characteristics of a main dominant wind wave field. No evident crossing sea characteristics of overlapping swell and wind components are observed. We have observed time-dependent wave statistics and proposed a novel approach to rationally analyze the non-stationary surface series.

The large wave characteristics measured do not exceed the conventional rogue thresholds^58^ *h*/*H*_*s*_ = 1.25 and *H*/*H*_*s*_ = 2.2 observed in laboratory experiments^15^. In contrast, Draupner, Andrea or Killard rogue waves^1,2^, all observed in intermediate water depths, did attain crest heights of approximately 1.6*H*_*s*_ (see Table 1). Nevertheless, our analysis reveals that the largest waves observed here have characteristics quite similar to those displayed by the El Faro, Andrea, Draupner and Killard rogue waves^1,2^ for which second order bound nonlinearities constitute the dominant factor enhancing the linear dispersive focusing of extreme waves.

Moreover, most observed values of the dimensionless depth *k*_*p*_*d* were slightly above (Doris) and below (Feb 2015) the threshold 1.363 above which unidirectional waves are expected to become modulationally unstable^30,31^. The sea states analyzed here were multidirectional, and a carrier wave is modulationally unstable even at depths below that critical value if they are perturbed by appropriate oblique disturbances^32–34^. This type of instabilities are not very likely to appear in theory^32^ if *k*_*p*_*d* < 0.5. Nonetheless, rogue waves can be generated by modulational instability, as in unidirectional seas^35,36^. However, in directional seas such as the two considered here, energy can spread directionally and the recurrence of large waves as observed in unidirectional seas is largely attenuated or suppressed^1,2^. Indeed, our statistical analysis indicates that modulational instabilities are ineffective, third-order resonant effects are negligible and second order bound nonlinearities are the dominant factor in shaping the large waves observed.

Our results here also indicate that in shallower water depths, nonlinear dispersion effects intensify^2,21^, inducing waves to break more rapidly than in deep waters. As a result, waves cannot breathe as they do not have time to grow and reach higher amplitudes above 1.25*H*_*s*_ as in deep water. Therefore, whereas the standard rogue thresholds are based on the Rayleigh law appropriate to linear non-breaking Gaussian seas, it makes sense to consider more realistic thresholds and models that account for wave breaking since the latter limits wave growth and impedes the occurrence of rogue waves^2,21,22^.

Finally, large waves with higher and sharper crests do not display any secondary maxima or minima. They appear more regular or “narrow banded” than relatively low waves, and their heights and crests do not often violate the Miche–Stokes type upper limits^59^. Our results also suggest that third-order resonant nonlinearities do not affect the surface statistics in any discernable way, in agremeent with recent rogue wave studies^1,2^. Indeed, our analysis reveals that fourth order cumulants are negligible. As a consequence, the sea states analyzed here have nothing specially rogue about them.

## Methods

### ADCP measurements

A Teledyne Sentinel V acoustic Doppler current profiler (ADCP) was deployed off Killard Point, Ireland (upper inset of Fig. 1) during Spring 2015 and off the Aran Islands, Ireland (lower inset of Fig. 1) during Spring 2017, to measure wave events. The instrument itself was secured in a frame to ensure it stayed in position and to prevent damage (see right panel of Fig. 1). The frame and instrument were placed at rest at the sea bottom, at an average depth of 37 m (Killard Point) and 45 m (Aran Islands). The four slant beams made a 25° angle with the vertical, so that at the surface, the maximum distance between beams was approximately 35 m (Killard Point) and 42 m (Aran). This ADCP operates by emitted sound pulses in five beams (four slanted and one vertical) and using the Doppler effect to measure the movement of sound scatterers such as plankton and small particulates, within these beams^60^. Each beam divides the water column into 38 bins, separated by 122 cm. Because of hardware limitations, data were sampled at 2 Hz similarly to standard wave measurements gathered at oil platforms^14^. Drawing on^61^, the sampling error on estimating crest and wave heights, the so-called quantization error^62,63^, is approximately 1–2%. This is mitigated by correcting for crest amplitudes by quadratically interpolating the sampled crests, as in recent stereo-measurements of the ocean surface^62,63^.

We correct the resulting data sets of echo intensity and velocity measurements for pitch, roll, and heading of the instrument in the water, and convert from instrument coordinates (a radial set) to geographical coordinates (North, East, Up) using standard transformations^64,65^. We interpolate the data to find the position of maximum intensity, corresponding to the location of the free surface^64^.

### Directional spectrum

We estimate the directional spectrum from the free-surface profiles of the four slanted beams using the DIWASP toolbox^66^. Consequently, we are able to determine angular spreading and directionality of a sea state. DIWASP uses a number of methods to estimate the directional spectrum from the cross-power spectrum of the data: direct Fourier transform method (DFTM), extended maximum likelihood method (EMLM), iterated maximum likelihood method (IMLM), extended maximum entropy principle (EMEP), and Bayesian direct method (BDM)^66^.

However, the estimations are not perfect due to limited information and unknown factors. The EMLM spectrum is often more directionally-diffused with a lower peak. The EMEP spectrum produces a good directional spreading. However, although the peak is higher than EMLM, it is below the desired result^67^. EMEP and BDM can give very similar spreading results, but their peak values often differ significantly^68,69^. EMEP can calculate bi-directionality, while BDM is less sensitive to probe layout and more robust against errors^68^. In our analysis, we consider the BDM spectrum.

### Wave parameters

The significant wave height *H*_*s*_ is defined as the mean value *H*_1/3_ of the highest one-third of wave heights. It can be estimated either from a zero-crossing analysis or more easily but approximately from the omnidirectional surface spectrum $S(f)={\int }_{0}^{2\pi }\,{S}_{d}(f,\theta )\,d\theta$ as *H*_*s*_ ≈ 4*σ*, where $\sigma =\sqrt{{m}_{0}}$ is the standard deviation of surface elevations, ${m}_{j}=\int S(f){f}^{j}{\rm{d}}f$ denotes spectral moments. Further, *S*_*d*_(*f*,*θ*) is the directional wave spectrum with *θ* as the direction of waves of frequency *f*, and the cyclic frequency is *ω* = 2*πf*. In this paper, we use the spectral-based estimate, which is 5–10% larger than the actual *H*_1/3_ estimated from the actual time series.

The dominant wave period *T*_*p*_ = 2*π*/*ω*_*p*_ follows from the cyclic frequency *ω*_*p*_ of the spectral peak and *T*_0_ is the observed mean zero-crossing wave period. For Gaussian seas, this is equal to 2*π*/*ω*_0_, with ${\omega }_{0}=\sqrt{{m}_{2}/{m}_{0}}$. The associated wavelength *L*_0_ = 2*π*/*k*_0_ follows from the linear dispersion relation ${\omega }_{0}=\sqrt{g{k}_{0}\,\tan \,{\rm{h}}({k}_{0}d)}$, with *d* the water depth. The ‘mean’ or central frequency *ω*_*m*_ of the spectrum is defined as *ω*_*m*_ = *m*_1_/*m*_0_ ^3^ and the associated mean period *T*_*m*_ is 2*π*/*ω*_*m*_. Theoretical NB steepness^3,49^ is defined as *μ*_*m*_ = *k*_*m*_*σ*, where *k*_*m*_ is the mean wavenumber corresponding to *ω*_*m*_ via the linear dispersion relation

$${\omega }_{m}=\sqrt{g{k}_{m}{Q}_{m}},\,\,{Q}_{m}=\,\tanh ({k}_{m}d).$$

The group velocity

$${c}_{g}={\omega ^{\prime} }_{m}=\frac{1}{2}{c}_{m}\{1+\frac{2{k}_{m}d}{\sinh (2{k}_{m}d)}\},\,\,{c}_{m}=\frac{{\omega }_{m}}{{k}_{m}},$$

where *ω*_*m*_^'^ is the first derivative of the mean frequency with respect to the wavenumber *k*_*m*_.

The spectral bandwidth *ν* = (*m*_0_*m*_2_/*m*_1_^2^ − 1)^1/2^ gives a measure of the frequency spreading. For unimodal directional spectra, as those analyzed in this study, the angular spreading ${\sigma }_{\theta }=\sqrt{{\int }_{0}^{2\pi }D(\theta ){(\theta -{\theta }_{m})}^{2}{\rm{d}}\theta }$, where the angular spreading function $D(\theta )={\int }_{0}^{\infty }{S}_{d}(\omega ,\theta ){\rm{d}}\omega /{\sigma }^{2}$ and ${\theta }_{m}={\int }_{0}^{2\pi }\,D(\theta )\theta {\rm{d}}\theta$ is the mean direction. In general, ${\omega }_{0}={\omega }_{m}\sqrt{1+{\nu }^{2}}$. Furthermore, we define *R* = *σ*_*θ*_^2^/2*ν*^2^ as a dimensionless measure of the directionality of a sea state^37,70^. In terms of *ω*_*m*_, *k*_*m*_ and *q*_*m*_ = *k*_*m*_*d* based on the spectral centroid, the directional factor is given by^40^

$${\beta }_{S}=8{(\frac{{c}_{g}}{{c}_{m}})}^{3}\frac{{Q}_{m}^{2}}{\Omega ^{\prime\prime} },$$

and the depth factor^40^

$${\alpha }_{S}=4{(\frac{{c}_{g}}{{c}_{m}})}^{2}\frac{{Q}_{m}}{\Omega ^{\prime\prime} }{X}_{nl}=\frac{1}{2}{X}_{nl}{(\frac{{c}_{g}}{{c}_{m}})}^{-1}\,{\beta }_{S},$$

where

$${\omega ^{\prime\prime} }_{m}=-\frac{g}{4{\omega }_{m}{k}_{m}{Q}_{m}}\Omega ^{\prime\prime} ,$$

and

$$\Omega ^{\prime\prime} ={[{Q}_{m}-{k}_{m}d(1-{Q}_{m}^{2})]}^{2}+4{({k}_{m}d)}^{2}{Q}_{m}^{2}(1-{Q}_{m}^{2})\mathrm{.}$$

The nonlinear interaction coefficient is^40^

$${X}_{nl}=\frac{9{Q}_{m}^{4}-10{Q}_{m}^{2}+9}{8{Q}_{m}^{3}}-\frac{1}{{k}_{m}d}\{1+\frac{{(2{c}_{g}-{c}_{m}/2)}^{2}}{{c}_{S}^{2}-{c}_{g}^{2}}\}+{X}_{st},$$

where ${c}_{S}=\sqrt{gd}$ is the phase velocity in shallow waters,

$${X}_{st}=\frac{{[{c}_{m}+{c}_{g}(1-{Q}_{m}^{2})/2]}^{2}}{{Q}_{m}({c}_{S}^{2}-{c}_{g}^{2})}\frac{R}{R+{\alpha }_{\omega }/2},$$

accounts for the wave-induced setdown^40^, and

$${\alpha }_{\omega }=\frac{{c}_{m}^{2}}{{c}_{g}^{2}}(1-\frac{{c}_{g}^{2}}{{c}_{S}^{2}}).$$

### Statistical parameters

The normalized covariance function of zero-mean surface displacement *η*(*t*) is defined as $\psi (\tau )=\overline{\eta (t)\eta (t+\tau )}/{\sigma }^{2}$. An alternative measure for the spectral bandwidth is given by the Boccotti parameter ψ^*^ = |*ψ*(τ^*^)|, which is the absolute value of the first minimum of *ψ* at *τ* = τ^*^ ^9^ and ${\ddot{\psi }}^{\ast }=\ddot{\psi }({\tau }^{\ast })$ the corresponding second derivative with respect to *τ*.

Skewness coefficient *λ*_3_ and excess kurtosis *λ*_40_ of the zero-mean surface elevation *η*(*t*) are given by

11 $${\lambda }_{3}=\overline{{\eta }^{3}}/{\sigma }^{3},\,{\lambda }_{40}=\overline{{\eta }^{4}}/{\sigma }^{4}-3\,.$$

Here, overbars imply statistical averages and *σ* is the standard deviation of surface elevations. Clearly, the wave steepness *μ* = *λ*_3_/3 ^6^ relates to the skewness coefficient *λ*_3_ of surface elevations. For third-order nonlinear (NB) random seas the excess kurtosis^37,38^

12 $${\lambda }_{40}={\lambda }_{40}^{d}+{\lambda }_{40}^{b}$$

comprises a dynamic component *λ*_40_^*d*^ due to nonlinear quasi-resonant wave-wave interactions^39^ and a Stokes bound harmonic contribution *λ*_40_^*b*^ ^41^. Drawing on^40^ and using parameters based on *ω*_*m*_, *k*_*m*_ and *q*_*m*_ = *k*_*m*_*d*, wave skewness and bound excess kurtosis for narrowband (NB) waves in intermediate water are given by

13 $${\lambda }_{3,NB}=6{\mu }_{m}(\alpha +\Delta ),\,{\lambda }_{40,NB}^{b}=24{\mu }_{m}^{2}(\beta +\gamma +2{(\alpha +\Delta )}^{2})=\frac{4}{3}{\lambda }_{3,NB}^{2}(1+\frac{\beta +\gamma }{2{(\alpha +\Delta )}^{2}}),$$

where

14 $$\alpha =\frac{3-{Q}_{m}^{2}}{4{Q}_{m}^{3}},\,\beta =\frac{24+3{(1-{Q}_{m}^{2})}^{3}}{64{Q}_{m}^{6}},\,\gamma =-\frac{{\alpha }^{2}}{2},\,\Delta =-\frac{1}{4}\frac{{c}_{S}^{2}}{{c}_{S}^{2}-{c}_{g}^{2}}[2\frac{1-{Q}_{m}^{2}}{{Q}_{m}}+\frac{1}{{q}_{m}}]+{\Delta }_{ST},$$

where

$${\Delta }_{ST}=\frac{1}{4}{c}_{g}\frac{{c}_{m}+{c}_{g}(1-{Q}_{m}^{2})}{({c}_{S}^{2}-{c}_{g}^{2})}\frac{R}{R+{\alpha }_{\omega }/2}$$

contributes positively to the wave induced setdown^40^ due to the directional spread of waves. Assuming that the linear crest heights (*ξ*_0_) scaled with surface rms are Rayleigh-distributed, the mean wave-induced setdown in the still water level is given by 〈*ξ*_0_^2^〉*μ*_*m*_Δ = 2*μ*_*m*_Δ, where brackets denote statistical average.

When Δ_*ST*_ = 0, some algebra shows that *λ*_3,*NB*_ is the same as the original Marthinsen-Winterstein formulation^49,71^, developed nearly three decades ago in the form:

15 $${\lambda }_{3,NB}=3{\mu }_{m}({D}_{1}+{D}_{2}),$$

where

16 $${D}_{1}=\frac{1}{2}\frac{4{c}_{g}/{c}_{m}-1}{{({c}_{g}/{c}_{m})}^{2}{T}_{m}-{k}_{m}d},\,{D}_{2}=\frac{\cosh ({k}_{m}d)[2+\,\cosh (2{k}_{m}d)]}{2{\sinh }^{3}({k}_{m}d)}.$$

The coefficients *D*_1_ and *D*_2_ arise from the frequency-difference and frequency-sum terms of second-order wave-wave interactions. Note that *D*_1_ = 2Δ and *D*_2_ = 2*α* exactly in unidirectional waves for which Δ_*ST*_ = 0. Unfortunately, Eq. (13) are not valid in relatively shallow water depths as second and third-order terms of the associated Stokes expansion can be larger than the linear term (see Eq. (A18) in^41^) because of the divergent nature of *α* and *β*. Thus, the relative validity of the preceding results essentially assumes the constraints *αμ*_*m*_ ≤ 1 and *βμ*_*m*_/*α* ≤ 1. These are satisfied for all seas states of both storms studied here.

### Miche-Stokes upper limit

In Gaussian seas, surface displacements and thus wave and crest heights have unbounded ranges. In reality, surface elevations are neither exactly Gaussian nor unbounded. And, the crest-to-trough height of a large wave whose steepness approaches the Stokes limiting steepness is unlikely to exceed an upper bound. For long-crested waves in transitional water depths, Miche^59^ approximated this upper bound as

17 $$\frac{{H}_{{\rm{l}}{\rm{i}}{\rm{m}}}}{\sigma }=\frac{2\pi }{7\sigma k}\tanh (kd),$$

where *σ* is the standard deviation of the sea state, *k* is the wavenumber and *d* the water depth. Following Tayfun^45^ the corresponding Miche-Stokes limit for crest heights is estimated as

18 $$\frac{{h}_{\mathrm{lim}}}{\sigma }=\frac{{H}_{\mathrm{lim}}/\sigma }{1+{\psi }^{\ast }}+\frac{{\lambda }_{3}}{6}{(\frac{{H}_{\mathrm{lim}}/\sigma }{1+{\psi }^{\ast }})}^{2}.$$

The Miche-Stokes limit can be rewritten as a function of wave period *T* via the linear dispersion relation. Finally, note that in realistic oceanic seas, nonlinear wave dispersion is effective in limiting the wave growth as a precursor to breaking^21–23^. Thus, in wave fields generated by intense storms, the onset of breaking can occur well below the preceding Miche-Stokes type upper bounds^22,24,25^.

### The Tayfun-Fedele model for crest heights

We define *P*(*ξ*) as the probability that a wave crest observed at a fixed point of the ocean in time exceeds the threshold *ξH*_*s*_. For weakly nonlinear nonlinear seas, this probability can be described by the third-order Tayfun-Fedele model^4^,

19 $${P}_{TF}(\xi )={\rm{\Pr }}[h > \xi \,{H}_{s}]=\exp (-8{\xi }_{0}^{2})[1+\varLambda {\xi }^{2}(4\,{\xi }^{2}-1)],$$

where *ξ*_0_ follows from the quadratic equation *ξ* = *ξ*_0_ + 2*μξ*_0_^2^. Here, *μ* = *λ*_3_/3 is the Tayfun steepness: it represents an integral measure of wave steepness and relates to second-order bound nonlinearities. The parameter Λ = *λ*_40_ + 2*λ*_22_ + *λ*_04_ is a relative measure of third-order nonlinearities expressed in terms of the fourth-order cumulants *λ*_*nm*_ of surface elevation *η* and its Hilbert transform $\hat{\eta }$^4^. In particular, ${\lambda }_{22}=\overline{{\eta }^{2}{\hat{\eta }}^{2}}/{\sigma }^{4}-1$ and ${\lambda }_{04}=\overline{{\hat{\eta }}^{4}}/{\sigma }^{4}-3$. In this study, Λ is approximated solely in terms of the excess kurtosis as Λ_appr_ = 8*λ*_40_/3. This approximation follows from the NB relations between cumulants^43,72^ *λ*_22_ = *λ*_40_/3 and *λ*_04_ = *λ*_40_, valid as the spectral bandwidth *ν* tends to zero. Numerical computations^1^ indicate that Λ ≈ Λ_appr_ with an error of about 3% in wave fields where second-order nonlinearities are dominant, in agreement with observations^35,73^.

For second-order seas, Λ = 0 and *P*_*TF*_ in Eq. (19) leads to the Tayfun wave-crest distribution^3,6^

20 $${P}_{T}(\xi )=\exp (-8{\xi }_{0}^{2}),$$

where *ξ* = *ξ*_0_ + 2*μξ*_0_^2^. For Gaussian seas, *ξ*_0_ = *ξ* since *μ* = 0 and Λ = 0, and *P*_*TF*_ reduces to the Rayleigh distribution

21 $${P}_{R}(\xi )=\exp (-8{\xi }^{2}).$$

Note that the Tayfun distribution represents an exact theoretical result for large second-order wave crest heights and it depends solely on the steepness parameter defined as *μ* = *λ*_3_/3^6^.

### The Forristall’s Weibull model for crest heights

The exceedance probability for crest heights is given by^8^

22 $${P}_{F}(\xi )={\rm{\Pr }}[h > \xi {H}_{s}]=\exp (-{(\xi /\alpha )}^{\beta }),$$

where *α* = 0.3536 + 0.2561*S*_1_ + 0.0800*U*_*r*_, *β* = 2 − 1.7912*S*_1_ − 0.5302*U*_*r*_ + 0.284*U*_*r*_^2^ for multi-directional (short-crested) seas. Here, *S*_1_ = 2*πH*_*s*_/(*gT*_*m*_^2^) is a characteristic wave steepness and the Ursell number *U*_*r*_ = *H*_*s*_/(*k*_*m*_^2^*d*^3^), where *k*_*m*_ is the wavenumber associated with the mean period *T*_*m*_.

### The generalized Boccotti and Tayfun models for crest-to-trough wave heights

The third-order nonlinear statistics for crest-to-trough wave heights is described in terms of the generalized Boccotti distribution^10^

23 $${P}_{B}(y)=\Pr [H > y\,{H}_{s}]=\frac{1+{\ddot{\psi }}^{\ast }}{\sqrt{2\,{\ddot{\psi }}^{\ast }(1+{\psi }^{\ast })}}\exp (-\frac{4\,{y}^{2}}{1+{\psi }^{\ast }})[1+\frac{\varLambda \,{y}^{2}}{1+{\psi }^{\ast }}(\frac{{y}^{2}}{1+{\psi }^{\ast }}-\frac{1}{2})],$$

and the Boccotti parameters^9^ ψ^*^ and ${\ddot{\psi }}^{\ast }$ are defined above in the section where statistical parameters are described. For Gaussian seas (Λ = 0), the original Boccotti^9^ model is recovered

24 $${P}_{B}(y)={\rm{\Pr }}[H > y\,{H}_{s}]=\frac{1+{\ddot{\psi }}^{\ast }}{\sqrt{2\,{\ddot{\psi }}^{\ast }(1+{\psi }^{\ast })}}\exp (-\frac{4\,{y}^{2}}{1+{\psi }^{\ast }}).$$

The Tayfun model for wave heights is given by^4,74^

25 $${P}_{T}(y)={\rm{\Pr }}[H > y\,{H}_{s}]=\sqrt{\frac{1+{r}_{m}}{2{r}_{m}}}(1+\frac{1-{r}_{m}^{2}}{64{r}_{m}{y}^{2}})\exp (-\frac{4\,{y}^{2}}{1+{r}_{m}}),$$

where *r*_*m*_ = *r*(*T*_*m*_/2) is the value of the envelope *r*(*t*) of the covariance *ψ*(*t*) at *t* = *T*_*m*_/2. Finally we note that as spectral bandwidth *ν* tends to zero, all three parameters ψ^*^, ${\ddot{\psi }}^{\ast }$ and *r*_*m*_ tend to unity, and the Boccotti and Tayfun distributions both reduce to the Rayleigh distribution given by

26 $${P}_{R}(y)=\exp (-2{y}^{2}).$$

## Data Availability

The datasets generated during and/or analyzed during the current study are available from the corresponding author on reasonable request.
